# Anti-Kv1.4 antibody-associated myocarditis refractory to maintenance immunosuppressive therapy: a case report

**DOI:** 10.1093/ehjcr/ytag819

**Published:** 2026-11-02

**Authors:** Tsukasa Kato, Ken Miura, Hiroyuki Watanabe

**Affiliations:** Department of Cardiology, Akita University School of Medicine, 1-1-1 Hondo, Akita City, Akita 010-8543, Japan; Department of Cardiology, Akita University School of Medicine, 1-1-1 Hondo, Akita City, Akita 010-8543, Japan; Department of Cardiology, Akita University School of Medicine, 1-1-1 Hondo, Akita City, Akita 010-8543, Japan

**Keywords:** Case report, Anti-Kv1.4 antibody, Autoimmune myocarditis, Immunosuppressive therapy

## Abstract

**Background:**

Anti-Kv1.4 antibody-associated myocarditis is an extremely rare condition and its optimal long-term management remains unclear.

**Case summary:**

A 66-year-old woman presented with myasthenia gravis-like symptoms, marked creatine kinase elevation, leukopenia, and hypogammaglobulinaemia. She developed acute myocarditis with ventricular arrhythmias and a reduced left ventricular ejection fraction. Intravenous immunoglobulin and corticosteroid therapy resulted in rapid clinical and cardiac improvement. During cautious tapering of oral prednisolone, she was readmitted with febrile neutropenia and subsequently developed progressive ventricular dysfunction and cardiogenic shock. Cardiac magnetic resonance imaging demonstrated extensive myocardial fibrosis, and autopsy revealed diffuse lymphocytic infiltration. Post-mortem testing confirmed positivity for anti-Kv1.4 antibodies.

**Discussion:**

This case highlights the potentially fatal course of suspected anti-Kv1.4 antibody-associated inflammatory myocardial disease despite an initial favourable response to immunosuppressive therapy. It illustrates the diagnostic and therapeutic challenges of distinguishing immune-mediated progression from other causes of myocardial injury and implies the need for individualized long-term management strategies.

Learning points

- Myocarditis occurring with myositis or myasthenia-like manifestations should raise suspicion of uncommon and potentially severe immune-mediated aetiologies.
- A broad diagnostic work-up, including testing for anti-striated muscle antibodies, may help identify clinically relevant phenotypes; however, the pathogenic role of anti-Kv1.4 antibodies remains uncertain.
- Serial cardiac troponin assessment is essential to monitor myocardial injury, evaluate response to immunosuppression, and guide treatment duration and tapering.

## Introduction

Myocarditis has infectious, toxic, and autoimmune causes.^1^ Anti-Kv1.4 antibodies are rare anti-striated muscle antibodies associated with myasthenia gravis, thymoma, and skeletal and cardiac muscle involvement. However, optimal management and long-term outcomes remain uncertain. We report a case of refractory anti-Kv1.4 antibody-associated myocarditis with myasthenia gravis—like symptoms, leukopenia, and hypogammaglobulinaemia.

## Summary figure

**Figure:**
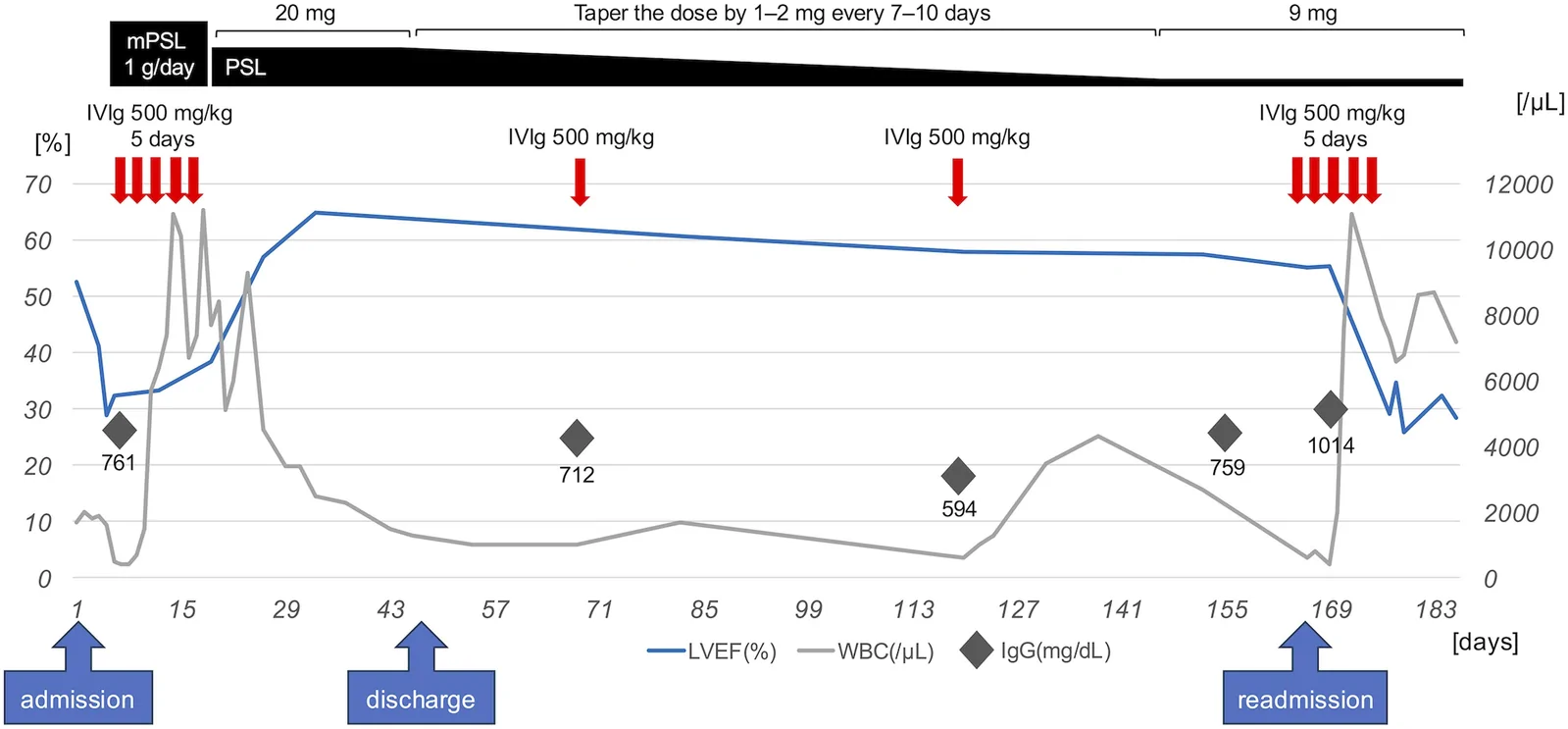
Changes in left ventricular ejection fraction, white blood cell count, serum immunoglobulin G levels, and treatment over time. The blue and grey lines indicate the left ventricular ejection fraction and white blood cell count, respectively, and the diamonds indicate the serum immunoglobulin G levels. Intravenous immunoglobulin administration is indicated by red arrows, and the oral prednisolone dose is shown above. The patient initially responded to intravenous immunoglobulin and corticosteroid therapy, with improvement in the left ventricular ejection fraction, but subsequently experienced a rapid decline during the second hospitalization while receiving maintenance prednisolone therapy. IgG, immunoglobulin G; IVIg, intravenous immunoglobulin; LVEF, left ventricular ejection fraction; mPSL, methylprednisolone; PSL, prednisolone; WBC, white blood cell count.

## Case presentation

A 66-year-old woman with no autoimmune disease, malignancy, regular medications, or prior immune checkpoint inhibitor therapy presented with diurnally fluctuating left ptosis and diplopia that had persisted for 2 weeks. Despite no myalgia or limb weakness, serum creatine kinase (CK) was 12 447 IU/L and high-sensitivity cardiac troponin T was 0.021 ng/mL. Although myasthenia gravis–associated myositis was suspected, anti-acetylcholine receptor and anti-muscle-specific tyrosine kinase antibodies were negative. She subsequently developed orthopnoea and cold extremities.

Electrocardiography revealed left axis deviation and ST-segment depression in leads I, aVL, and *V*_5–6_ (*Figure 1A*). Cardiac monitoring revealed frequent non-sustained ventricular tachycardia (*Figure 1B*). CK increased to 16 489 IU/L, with CK-MB of 388 IU/L and brain natriuretic peptide of 1050 pg/mL. Leukopenia was present (1700/μL; neutrophils, 730/μL; eosinophils, 190/μL; monocytes, 270/μL; lymphocytes, 460/μL), with reduced serum IgG and IgA. HIV testing was negative. Echocardiography showed a left ventricular ejection fraction (LVEF) of 40% and trace pericardial effusion (*Figure 1C*). Cardiac magnetic resonance (CMR) was precluded by haemodynamic instability. Computed tomography showed no thymoma (*Figure 1D*). Coronary angiography revealed no significant coronary stenosis, and right ventricular septal biopsy demonstrated dense lymphocytic infiltration and myocyte necrosis without multinucleated giant cells (*Figure 1E*), confirming acute myocarditis. Viral and autoimmune serology showed no acute infection or autoimmune disease. Bone marrow aspiration showed normocellular marrow without haematological malignancy.

**Figure 1:**
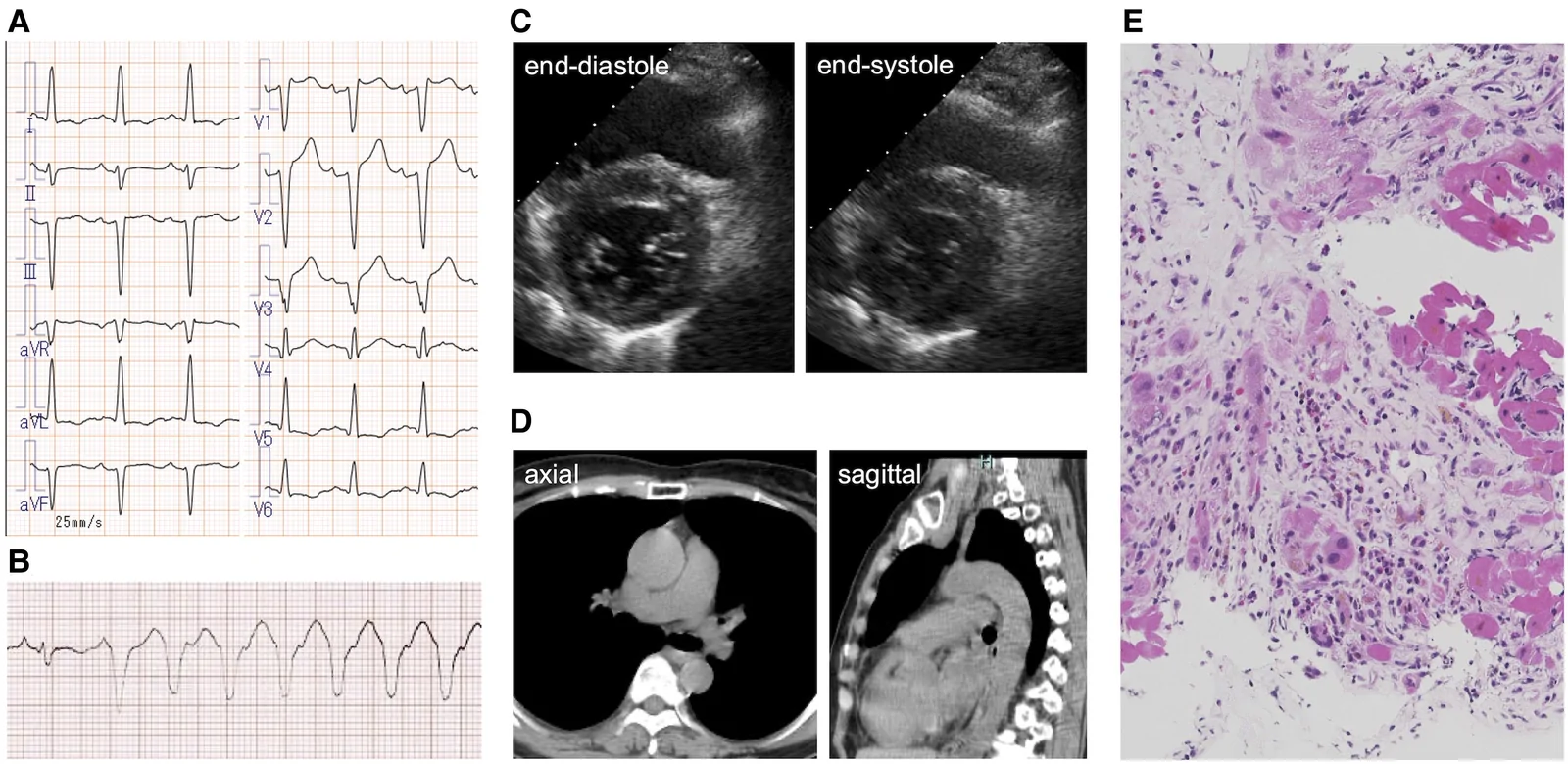
Electrocardiographic, echocardiographic, computed tomographic, and histopathological findings at initial hospitalization. (*A*) Standard 12-lead electrocardiogram on admission showing left axis deviation, poor R-wave progression in precordial leads *V*_1–3_, and ST-segment depression in leads I, aVL, and *V*_5–6_, with a QTc of 478 ms. (*B*) Continuous cardiac monitoring demonstrating non-sustained ventricular tachycardia. (*C*) Transthoracic echocardiography showing diffuse myocardial wall thickening and a reduced left ventricular ejection fraction of 40%, with trace pericardial effusion. (*D*) Axial and sagittal chest computed tomography images showing no evidence of thymoma or anterior mediastinal mass. (*E*) Histopathological analysis of a right ventricular septal biopsy specimen revealing dense lymphocytic infiltration and myocyte necrosis, consistent with acute myocarditis (haematoxylin–eosin staining).

Ventricular tachycardia was controlled with amiodarone. Despite decreasing CK, LVEF declined to 30% and hypercapnic respiratory failure progressed, necessitating mechanical ventilation and inotropic support. Myocarditis with rhabdomyolysis and myasthenia gravis-like symptoms suggested a possible autoimmune process associated with anti-striated muscle antibodies. Persistent leukopenia, despite granulocyte colony-stimulating factor, and hypogammaglobulinaemia suggested immune dysfunction. Lymphocyte subset analysis revealed a low CD19-positive B-cell fraction, increased CD8-positive T-cell fraction, and a reduced CD4/CD8 ratio.

Intravenous immunoglobulin (IVIg; 500 mg/kg/day for 5 days) and intravenous methylprednisolone (1 g/day for 3 days) rapidly restored LVEF to 65% and resolved the neurological symptoms. Maintenance oral prednisolone was initiated at 20 mg/day (0.5 mg/kg). After acute-phase activity restriction, supervised low-intensity rehabilitation progressed to 500-m walking.

Prednisolone was tapered to 9 mg/day over 4 months and continued until readmission. LVEF remained preserved and CK-MB normalized, but cardiac troponin was not serially assessed thereafter; therefore, persistent or recurrent myocardial injury during maintenance corticosteroid therapy could not be adequately evaluated. During follow-up, periodic IVIg was empirically administered for recurrent hypogammaglobulinaemia with leukopenia, primarily to reduce infection risk.

The patient was readmitted with febrile neutropenia. Electrocardiography showed complete right bundle branch block, left axis deviation, and ST-segment elevation in leads *V*_3–4_ (*Figure 2A*). CK remained normal, and neurological symptoms were absent. Although LVEF was initially preserved, it rapidly declined to 32% within 10 days. Echocardiography revealed left ventricular wall thinning and an enlarged end-diastolic diameter of 60 mm, mimicking dilated cardiomyopathy (*Figure 2B*). CMR demonstrated circumferential predominantly epicardial late gadolinium enhancement (LGE) (*Figure 2C*) and scattered low-signal intensity on T2-weighted imaging (*Figure 2D*), suggesting chronic myocardial fibrosis and remodelling rather than active oedema. Repeat viral serology and blood-based herpesvirus multiplex polymerase chain reaction showed no evidence of acute viral infection; repeat autoimmune serology was unrevealing. Although infection could not be completely excluded, microbiological evidence was absent.

**Figure 2:**
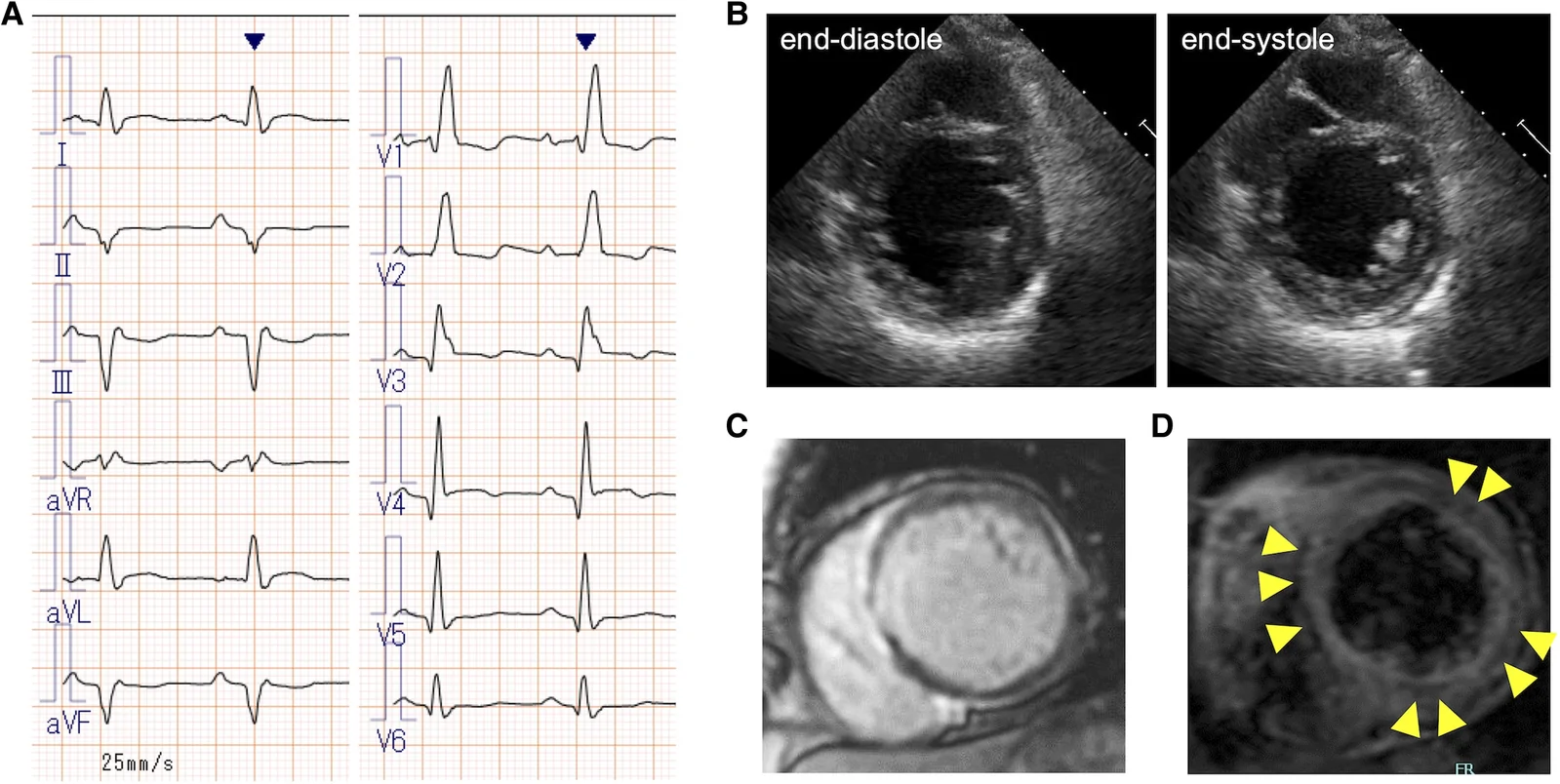
Electrocardiographic, echocardiographic, and cardiac magnetic resonance findings at the second hospitalization. (*A*) Standard 12-lead electrocardiogram showing sinus rhythm with complete right bundle branch block, left axis deviation, and ST-segment elevation in leads *V*_3–4_, with a QTc of 465 ms. (*B*) Transthoracic echocardiography showing thinning of the left ventricular wall, an increased end-diastolic diameter, and a severely reduced left ventricular ejection fraction of 32%. Cardiac magnetic resonance imaging demonstrating (*C*) circumferential late gadolinium enhancement predominantly in the epicardial layer along with (*D*) scattered areas of low-signal intensity on T2-weighted imaging (yellow arrowheads).

The patient subsequently developed cardiogenic shock unresponsive to intra-aortic balloon pumping, continuous haemodialysis, and mechanical ventilation. She died of multi-organ failure within days. The clinical course is summarized in the *Summary Figure*.

Autopsy revealed extensive lymphocytic infiltration with associated myocyte injury and scattered fibroblasts in the left ventricular myocardium, consistent with ongoing myocardial inflammation and chronic remodelling (*Figure 3A–D*). Post-mortem analysis of stored serum samples demonstrated positivity for anti-Kv1.4 antibodies. Although imaging and autopsy did not reveal a thymoma, the coexistence of leukopenia, lymphopenia, and hypogammaglobulinaemia implied an underlying immune dysregulation.

**Figure 3:**
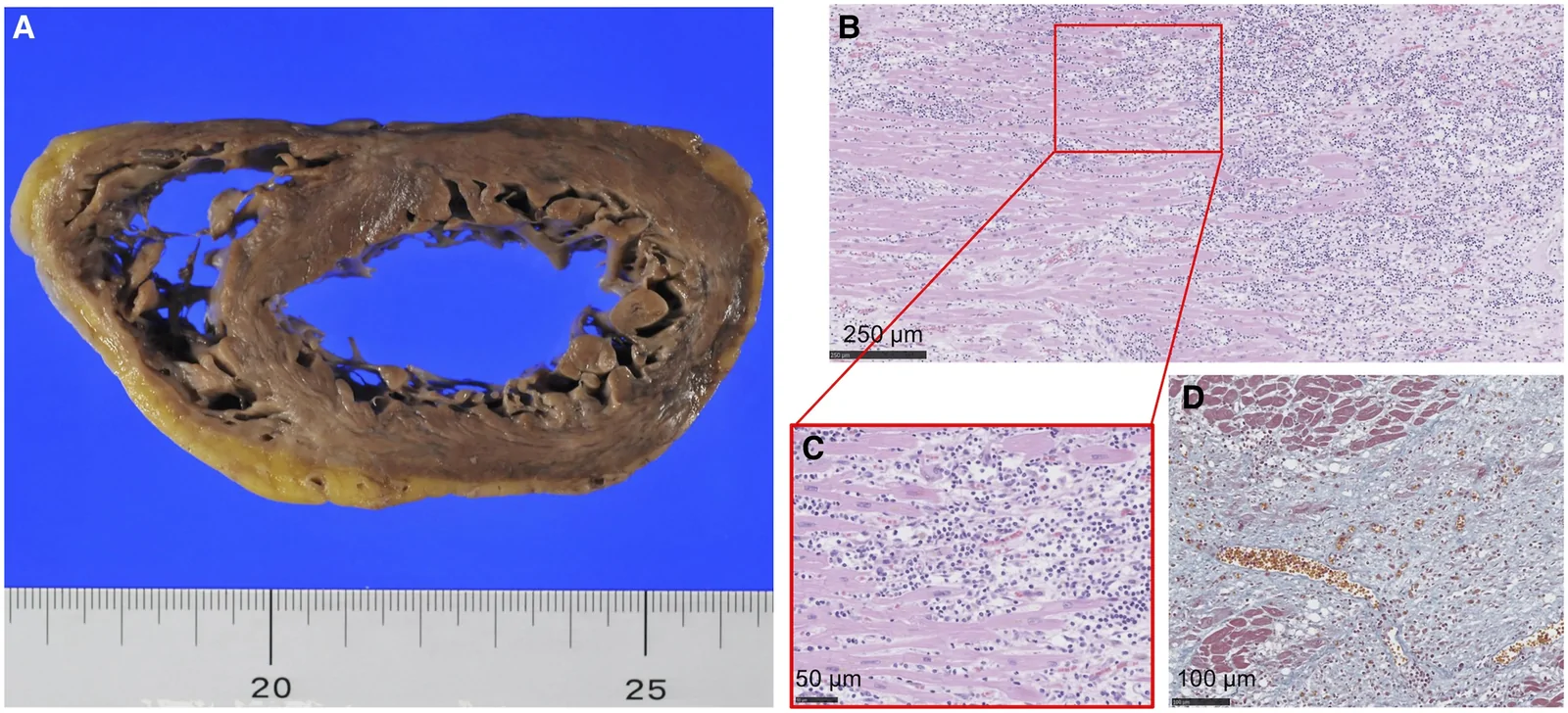
Gross and histopathological autopsy findings. (*A*) Gross examination of the heart showing extensive myocardial discoloration and thinning of the left ventricular wall. (*B*) Low-power view (haematoxylin–eosin staining) demonstrating dense lymphocytic infiltration throughout the myocardium. (*C*) High-power view (haematoxylin–eosin staining) showing extensive lymphocytic infiltration with associated myocyte injury. (*D*) Elastica–Masson staining revealing scattered fibroblasts suggestive of early fibrotic remodelling.

## Discussion

This report illustrates a rare and diagnostically challenging case of suspected anti-Kv1.4 antibody-associated myocarditis. The pathogenic role of anti-Kv1.4 antibodies remains unproven; they may represent a serological marker of autoimmune myocarditis and myositis rather than a direct mediator of myocardial injury.^2,3^ The patient presented with myasthenia gravis—like symptoms and markedly elevated CK despite the absence of limb weakness. Kuyama *et al*.^2^ similarly described anti-Kv1.4 antibody-positive myocarditis without myasthenia gravis or thymoma, suggesting a broader clinical spectrum. Although IVIg and corticosteroids achieved transient improvement, the disease proved refractory and fatal despite maintenance prednisolone therapy.

The 2025 European Society of Cardiology (ESC) Guidelines emphasize aetiology- and risk-based management of myocarditis.^4^ For virus-negative lymphocytic myocarditis, corticosteroids and, in selected cases, additional immunosuppressive agents, may be considered. Previous reports have suggested that adding calcineurin inhibitors to corticosteroids may be beneficial in treating anti-Kv1.4 antibody-positive myocarditis, although long-term outcomes and optimal intensity and duration of immunosuppression remain unclear.^2^ In our case, earlier treatment intensification might have been considered; however, anti-striational antibodies were not tested during the initial episode because the assays were not routinely available and anti-striational antibody-associated disease was not recognized. In retrospect, fluctuating ocular symptoms, severe myositis, and myocarditis despite negative conventional myasthenia gravis–associated antibodies should have prompted testing. Anti-titin and other anti-muscle antibodies have also been reported in patients with myasthenia gravis and overlapping myositis and myocarditis.^5,6^ Post-mortem testing detected anti-Kv1.4 but not anti-titin antibodies, although other relevant autoantibodies could not be excluded. Thymectomy was not pursued because computed tomography showed no thymoma and myasthenia gravis was not definitively diagnosed; autopsy confirmed absence of thymoma. Profound leukopenia and hypogammaglobulinaemia also increased the risk of infection and limited the feasibility of intensified immunosuppression.

During the subsequent hospitalization, immunosuppression was not immediately intensified because febrile neutropenia raised concern for infection and the presentation differed from the initial episode. CMR showed extensive LGE without T2 hyperintensity, with left ventricular dilation and wall thinning, favouring chronic fibrosis and remodelling over recurrent active myocarditis. Consequently, repeat endomyocardial biopsy was not performed. In retrospect, repeat biopsy should have been considered because persistent inflammation could not be excluded, particularly in the absence of serial cardiac troponin measurements. This approach would have been consistent with the 2025 ESC Guidelines, which recommend considering biopsy when its findings may influence diagnosis or treatment.^4^ Confirmation of active lymphocytic myocarditis might have supported additional immunosuppression, but its potential benefit had to be weighed against the substantial risk of infection associated with leukopenia, hypogammaglobulinaemia, and febrile neutropenia. The predominantly epicardial LGE and ventricular remodelling also overlapped with imaging phenotypes of desmoplakin- or filamin C-related cardiomyopathy^7^; because genetic testing was not performed, an underlying genetic susceptibility could not be excluded.

Persistent leukopenia, comprising neutropenia and lymphopenia, and hypogammaglobulinaemia were present before corticosteroid therapy. Evaluations did not support HIV infection, bone marrow failure, haematologic malignancy, or thymoma-associated immunodeficiency. These findings suggested unexplained immune dysregulation that may have contributed to the severe clinical course.

## Conclusions

This case highlights the diagnostic difficulty of suspected immune-mediated myocarditis, emphasizing the need for broad evaluation with anti-striated muscle antibody testing and serial cardiac troponin assessment to guide immunosuppressive therapy.

## Lead author biography


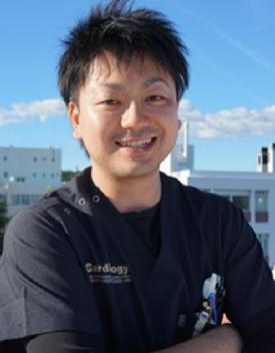


Tsukasa Kato graduated from YamagataUniversity School of Medicine in 2012. He is currently a cardiologist at AkitaUniversity Hospital. He is specializingin cardiac catheterization.

## Data Availability

All data relevant to this case report are included in the article. No additional datasets were generated or analysed.
